# Supplementary figures and images for: Offline prompt reinforcement learning method based on feature extraction
Source: PeerJ Comput Sci. 2025 Jan 2;11:e2490. doi: 10.7717/peerj-cs.2490 (PMC11784719; doi:10.7717/peerj-cs.2490)

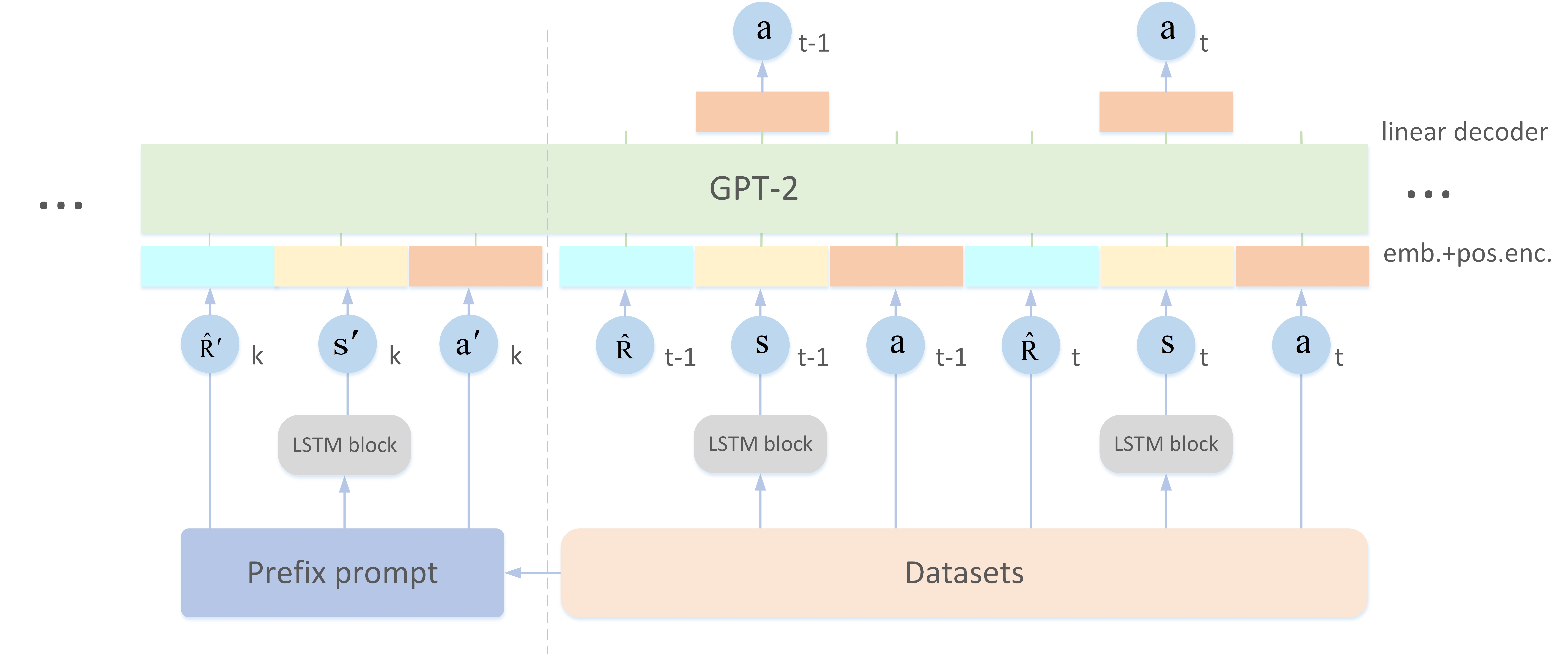

Supplement: Supplemental Information 1 — Algorithm source code [file peerj-cs-11-2490-s001.zip › PLDT-main/architecture.jpg]
